# Risky business: COVAX and the financialization of global vaccine equity

**DOI:** 10.1186/s12992-021-00763-8

**Published:** 2021-09-20

**Authors:** Felix Stein

**Affiliations:** Postdoctoral Researcher, Centre for Development and the Environment (SUM), University of Oslo, Sandakerveien 130, 0484 Oslo, Norway

**Keywords:** COVAX, COVID-19, Risk, Finance, Financialization, Vaccines

## Abstract

**Background:**

During the first year and a half of the COVID-19 pandemic, COVAX has been the world’s most prominent effort to ensure equitable access to SARS-CoV-2 vaccines. Launched as part of the Access to COVID-19 Tools Accelerator (Act-A) in June 2020, COVAX suggested to serve as a vaccine buyers’ and distribution club for countries around the world. It also aimed to support the pharmaceutical industry in speeding up and broadening vaccine development. While COVAX has recently come under critique for failing to bring about global vaccine equity, influential politicians and public health advocates insist that future iterations of it will improve pandemic preparedness. So far COVAX’s role in the ongoing *financialization* of global health, i.e. in the rise of financial concepts, motives, practices and institutions has not been analyzed.

**Methods:**

This article describes and critically assesses COVAX’s financial logics, i.e. the concepts, arguments and financing flows on which COVAX relies. It is based on a review of over 109 COVAX related reports, ten in-depth interviews with global health experts working either in or with COVAX, as well as participant observation in 18 webinars and online meetings concerned with global pandemic financing, between September 2020 and August 2021.

**Results:**

The article finds that COVAX expands the scale and scope of financial instruments in global health governance, and that this is done by conflating different understandings of risk. Specifically, COVAX conflates public health risk and corporate financial risk, leading it to privilege concerns of pharmaceutical companies over those of most participating countries – especially low and lower-middle income countries (LICs and LMICs). COVAX thus drives the financialization of global health and ends up constituting a risk itself - that of perpetuating the downsides of financialization (e.g. heightened inequality, secrecy, complexity in governance, an ineffective and slow use of aid), whilst insufficiently realising its potential benefits (pandemic risk reduction, increased public access to emergency funding, indirect price control over essential goods and services).

**Conclusion:**

Future iterations of vaccine buyers’ and distribution clubs as well as public vaccine development efforts should work towards reducing all aspects of public health risk rather than privileging its corporate financial aspects. This will include reassessing the interplay of aid and corporate subsidies in global health.

## Background

### COVAX and global health

COVAX is an alliance of various established global health institutions that aims to improve worldwide access to vaccines against COVID-19. Its original goal was to deliver at least 2bn vaccine doses to countries around the globe by the end of 2021. Headed by Gavi, the Vaccine Alliance, the Coalition for Epidemic Preparedness Innovations (CEPI) and the World Health Organization (WHO), COVAX was promoted as “the only truly global solution to the pandemic because it is the only effort to ensure that people in all corners of the world will get access to COVID-19 vaccines [ …], regardless of their wealth” [1]. Indeed, in terms of funding, power and media attention, COVAX has been the most important global health effort during the first year of the COVID-19 pandemic. It was first announced in March 2020 by leaders of the world’s most affluent economies (the G20), who argued in an official statement that they were determined to fight the pandemic and to work with the private sector [2]. This culminated in unveiling a new public private partnership (PPP) on 24 April 2020 called “The Access to COVID-19 Tools Accelerator” (Act-A), of which COVAX is the vaccines pillar.

Act-A and COVAX continue established trends in global health governance such as a disease-specific i.e. “vertical” approach to health financing and care, a focus on health technologies, and a penchant for market solutions to tackle public health issues [3, 4]. Thus, one of Act-A’s founding documents begins by arguing that non-pharmaceutical interventions against the pandemic, based on behavioural change are ineffective. They may flatten the curve of new infections, but they “come at an enormous cost – effectively freezing social and economic life around the world”, allegedly without addressing “the root causes of the crisis” [5]. As Act-A largely precludes engagement with the structural causes and catalysts of COVID-19 [6], it instead focuses the fight against the pandemic around three sets of technologies, namely diagnostics, treatments and vaccines. Together these technologies constituted 75% ($28,6bn) of Act-A’s initial target budget of $38,1bn [7, 8], greatly outweighing work on health systems, which originally also lacked an investment case [9, 10]. COVAX, as Act-A’s vaccine pillar, is by far the most important of the three sets of technologies. It made up 42% ($16bn) of Act-A’s initial target funding (ibid) and as of mid-August 2021 it has received around 70% ($12,5bn) of all allocated funding pledges to Act-A ($18bn) [8]. It is currently the only pillar of Act-A that has exceeded its (recently revised) target funding requirements ($11.7bn for 2021) [8, 9], far outperforming Act-A’s other spheres of activity.

Yet, COVAX does not merely continue the search for private, marketable solutions to global health problems, solutions that have come to shape ‘market multilateralism’ and neoliberal healthcare in recent decades [11]. Instead, an analysis of COVAX’s financial logics suggests that it expands the scale and scope of *financial* instruments in global health governance, and that this is done by conflating different understandings of “risk”.

### The financialization of global health

Global health has in recent decades become increasingly financialized, meaning it has become oriented more and more towards financial concepts, motives, practices and institutions [12]. While the financialisation of global health accompanies the rise of market mechanisms and neoliberal approaches to health, it also differs from these trends, in that it constitutes a mode of capital accumulation in its own right, one based on creating and re-creating monetary debt relationships [13–15]. Financial capital accumulation focuses on making money primarily by using existing money, contracts and time. Its rise has a profound influence on how both public and private health entities operate. This has been shown for pharmaceutical companies and the producers of ventilators for example, whose strategic interests shift away from producing medical goods and services and towards securing income and shareholder value through financial means [16, 17].

Key actors that drive the financialization of global health governance include COVAX’ governing institutions Gavi and CEPI; the World Bank, which partners with COVAX on vaccine financing and research; and the Bill and Melinda Gates Foundation, which has been instrumental in developing COVAX in the first place [18]. United in the belief that private solutions are generally preferable to public sector ones, these institutions tend to create more financial markets in health, to respond to the twofold problem of a lack of global public health funding and a worldwide excess in private capital [19, 20].

The World Bank, has been at the forefront of this trend, by issuing the first vaccine bonds together with Gavi in 2006 and by creating the Pandemic Emergency Financing Facility (PEF) in 2017. The PEF was an insurance that opened pandemic preparedness to private investment, offering investors substantial returns from public coffers for betting against disease outbreaks [21, 22]. Both Bank initiatives illustrate several of the potential downsides of financialization as they turned out to be highly complex and thus poorly understood by potential beneficiaries, the aid community, and in part even by the people putting them together [23, 24]. The PEF was also costly, not just because its pay-out triggers favoured investors rather than the organisations and governments whom it was meant to cover [25, 26] but also because the private sector tends to have a higher cost of capital than high income countries [22]. While the COVID-19 pandemic wiped out the hope of further PEF-related profits for private investors, the World Bank continues to work on a PEF 2.0 [27].

Another institution driving the financialization of global health is the Gates Foundation, which in the mid-2000s began to complement traditional health programme financing, with financing efforts that provide returns to private investors [28, 29]. While this has made large amounts of private sector capital available for global health, it has also contributed to rendering global health governance more secretive than it had previously been, driven by the Foundation’s reliance on private equity funds, whose expansion in developing countries it actively encourages [29, 30]. Such efforts also stand in tension to empirical studies that show how overly financialized healthcare increases inequity [15, 19]. The heavy involvement of private equity funds in healthcare in Turkey for example, has lead hospitals to offer increasingly different standards of care to patients based on their ability to pay [31].

Yet, the financialization of global health is not just driven by institutions, but also by changes in health discourse. In fact, discourse is more important for the world of finance than for other modes of capital accumulation (such as manufacturing or trade) because finance itself is largely conceptual and linguistic in nature [32, 33]. The debt relationships on which financial activity relies continuously need to be described and justified to lead to economic growth. A well-documented discursive shift aiding the financialisation of healthcare, was the publication of the World Bank’s 1993 report, entitled “Investing in Health”. It described human health not primarily as a goal in and of itself, but presented it as an object of “human capital investment”, a notion that has since culminated in the publication of the Bank’s Human Capital Index [34].

This article argues that COVAX adds another discursive push in favour of the financialisation of global health, by amalgamating two different understandings of “risk”. As a PPP that combines actors at unforeseen scale and scope [35] COVAX relies on the idea of public health risk as a diplomatic tool, to mediate between different groups of actors with strongly diverging interests: Rich and poor countries who have been historically at odds with one another over vaccine equity and other health issues are told that they share the same public health risk and should therefore cooperate. Here the term risk refers to “factors that raise the probability of adverse health outcomes” [36], which are increasingly important in industrialised society [37] and which can be attenuated by institutions of the welfare state for example [38]. At the same time, however, COVAX invokes the idea of risk to refer to various forms of corporate and financial risk, faced by pharmaceutical companies. It mainly uses financial tools to alleviate corporate financial risk, an approach to public health that was developed by Gavi, the World Bank and the Gates Foundation to support developing countries’ vaccine access but that has now been expanded to the world at large. In amalgamating both kinds of risk, COVAX ends up constituting a risk in itself [39]. It may perpetuate the downsides of the financialisation of global health, such as heightened inequality, secrecy and complexity in governance, and an ineffective and slow use of aid, whilst insufficiently realising its potential benefits, namely actual pandemic risk reduction, an increased public access to emergency funding and indirect price control over essential goods and services.

## Methods

This paper is part of a research project based at the Centre for Development and the Environment at the University of Oslo and funded by Research Council of Norway (grant number 301929), which studies new forms of cooperation between public and private actors in pandemic preparedness. It draws on three sources that provide qualitative insight into COVAX’s financial logics and that complement one another. These are a review of COVAX-related grey literature, ten in-dept interviews with global health experts involved in setting up and working with different parts of COVAX, as well as participant observation in 18 webinars and online meetings either directly concerned with COVAX or with pandemic financing in times of COVID-19 more generally.

### Grey literature

I have reviewed all available official reports published by four of COVAX’s leading institutions in their online report repositories. These include 54 reports published between April 2020 and August 2021 on the WHO’s document repository under the heading “ACT Accelerator” [40], 37 reports published between June 2020 and August 2021 on the same repository under the heading “COVAX” [41], 24 reports published between April 2020 and August 2021 on GAVI’s “COVAX Facility” website [42], and 4 reports published between December and October 2020 in CEPI’s “Document Library” [43]. I downloaded all 119 documents, discarded 10 duplicates, and read the remaining 109 documents with a focus on COVAX financing. This body of grey literature was complemented by reading the websites and news releases of COVAX’s leading institutions (notably Gavi, CEPI and WHO). Given that the majority of COVAX’s self-descriptions in the public domain are sales pitches that stand in the service of fundraising and building political legitimacy, I have put them under critical scrutiny by reviewing press and scholarly analyses of COVAX published between April 2020 and August 2021. I also followed Susan Erikson’s method of paying special attention to the monetary flows that COVAX engenders. Considering these flows to be signifiers of value enables them to serve as a reality check against promotional rhetoric [29].

### Interviews

A second set of sources used to make sense of COVAX’s financial logics are ten key informant interviews conducted between September 2020 and August 2021 with health and policy makers involved in its creation or working closely with COVAX. Interviewees included members of COVAX’s governing institutions, academics involved in creating and assessing it and government ministers working with ACT-A. They were contacted via email interviewed via video-call for around 1 hour each and recorded with their consent. I conducted 8 of the interviews myself and members of my team two additional ones. Interviews were subsequently anonymized and transcribed verbatim. They provide additional insight into the history of COVAX’s creation and foreground some of the motivations, concerns and institutional alternatives that continue to shape its evolving structure and ways of operating.

### Participant observation in webinars and civil society consultations

Finally, this article draws on participant observation in 18 global health financing webinars between September 2020 and August 2021, as well as a roundtable discussion about Norway’s proposed principles for pharma action, held on February 19, 2021 with representatives from four Norwegian civil society organisations as well as academics involved in health equity research and advocacy [44]. These online meetings and conferences elucidated how COVAX works in detail, provided contextual information that foregrounds policy alternatives to it, and highlighted reactions of civil society representatives to COVAX’s health equity approach.

## Results

### A global buyers’ and distribution club

COVAX was originally created to bring about global “vaccine equity”, a fair distribution of vaccines, which can be considered a technology-based subset of and a substitute for health equity. As one interviewee who was invited to work with CEPI and the World Bank on COVAX financing in early 2020 put it in an interview:

“A lot of world leaders were saying [at the beginning of the outbreak] that we needed to turn [COVID-19 vaccines] into a global public good. [French President Emmanuel] Macron and many others recognized that we could not make the same mistakes as we had done during the 2009 H1N1 pandemic, where rich countries had struck bilateral deals with vaccine manufacturers and had gotten more vaccines earlier. [ … ] That had been a moral catastrophe that prolonged the pandemic so we would do things differently”.

A background paper for COVAX from March 2020 confirms equity as the institution’s main goal:

“Affordability and accessibility must be the bedrock of any proposal for a new funding push for COVID-19 vaccine development. The poor are hit first and worst by outbreaks, and any access model that ends up giving only high-income countries access to the vaccine would clearly be unacceptable. It will be critical to avoid a scenario in which high-income country governments enter into bilateral purchase contracts with manufacturers, thus monopolizing the vaccine” [27].

One main way in which COVAX was meant to pursue its goal was by establishing a global buyers’ and distribution club in which Gavi would purchase vaccines on behalf of all member states and enable them together with UNICEF and PAHO to share vaccine doses equitably among one another [45]. COVAX suggested distributing vaccines in two phases. In a first phase it would distribute doses proportionally, first covering 3% of member countries’ populations, to protect health and social care workers and thereafter covering up to 20% to inoculate most high-risk adults. In a second phase, doses would be allocated based on more vaguely defined COVID-19 threat and country vulnerability criteria, with the help of proprietary algorithms [46]. While this global vaccine allocation model has been criticized for being overly pragmatic and imprecise [47] and for capping explicit distributive targets at 20% of the world’s population, policy makers tend to agree that it is still preferable to unfettered market competition.

According to my interviewees, one of the main difficulties that COVAX faced was to convince wealthy countries to join in (see also [48, 49]). However, a key utilitarian advantage meant to appeal to wealthy countries lay in the buyers’ club’s potential for risk reduction. Firstly, global health collaboration itself was presented in terms of public health risk. Based on the slogan “nobody is safe until everybody’s safe” COVAX representatives continue to insist that with respect to COVID-19, the health of citizens in the global north and in the south are closely and irrevocably intertwined [50]. Countries in the global north may have been able to ignore far away health concerns and even infectious disease outbreaks in the past without severely compromising their citizens’ health, yet the highly infectious and often asymptomatic nature of Sars-CoV-2 and the possibility of it developing more and more aggressive variants are held to make this impossible today. Global collaboration in a single PPP is here considered necessary and framed as relatively cheap, when compared to the pandemic’s potential economic costs and to the public spending on the pandemic so far [51]. This invocation of a uniformly shared health risk can be seen as part of an established global health security discourse that has long framed infectious disease risk as an outside threat to national health and economic growth [52].

In addition, COVAX also aimed to reduce the risk for countries that individual vaccine companies would fail to produce a viable product. The buyers’ club was a hedge against potential corporate failure. As the chief executive officer of Gavi underlined early on:

“There are currently more than 170 candidate vaccines in development, but the vast majority of these efforts are likely to fail. ( … ) To increase the chances of success, COVAX has created the world’s largest and most diverse portfolio of these vaccines” [1].

Corporate vaccine development risk could be reduced by relying on the expertise of CEPI which actively manages COVAX’s vaccine portfolio with the help of its research and development experts [53]. COVAX thus promised to ensure that its vaccine candidates would rely on different kinds of technologies and that they would span various geographies. Such risk reduction efforts would eventually experience a major setback, when India’s vaccine export stop exposed the world’s (and COVAX’s) overreliance on the Indian Serum Institute. However, Important for this article is that COVAX presented corporate failure as a priority for public health governance and corporate risk as a direct extension of public health risk.

The buyers’ club promised the third practical advantage of exerting increasing price control over vaccine companies. As one COVAX report puts it “Pooling risks not only means a greater chance at shared rewards through access to successful vaccine candidates, it also means lower prices as competition in a non-pooled risks scenario leads to a disorderly market with price gouging [ …]” [54]. COVAX would reduce competition and information asymmetries between member countries and use their combined purchasing power to hold prices at minimal level during the pandemic. Covax supporters hoped that that vaccine manufacturers would “provide a “cost plus” contract with a small profit margin during the acute phase of the pandemic [27]. If COVID-19 was to become a globally endemic pathogen, successful vaccines could eventually transition to commercial pricing [27].

The idea of a shared public health risk that included vaccine production risks thus served as the conceptual glue meant to hold the global vaccine buyers’ club together. However, as part of a series of concessions, mostly to high income countries (HICs), the buyers’ club was weakened internally to the point of becoming dysfunctional and the idea of globally shared public health risk was compromised. Firstly, COVAX enabled its members to strike bilateral vaccine deals on the side (contrary to the European Union’s vaccine buyers’ club for example). This concession greatly weakened COVAX’ structure, as the PPP had initially been set up precisely to avoid an international bilateral scramble for vaccines. COVAX facilitated bilateral deals even further by conceding to pressure from the United Kingdom [49] and providing members with the option to commit only to a select sub-section of its vaccine portfolio. Such ‘Optional Purchase Agreements’ it was argued “may be more attractive to participants that already have bilateral agreements with manufacturers through which they may already have secured sufficient doses of that particular vaccine” [1]. As a result, COVAX was reduced from a buyer’s club to a mere “insurance policy” for affluent countries that might buy vaccines elsewhere [1].

**Fig. 1:**
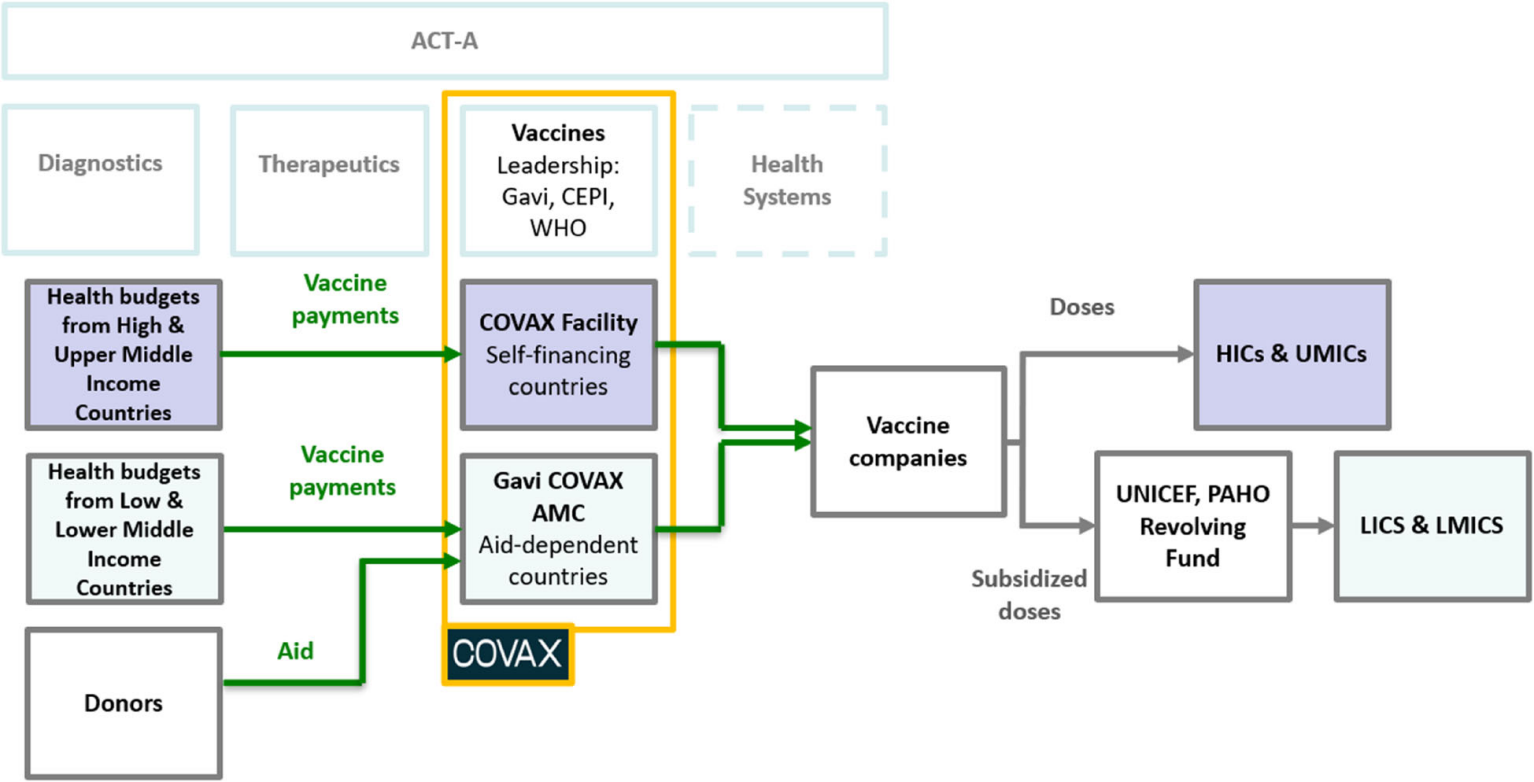
COVAX was split into two buyers’ and distribution clubs. Based on [54, 55]

To further cater to the world’s most affluent economies, COVAX member contributions were not directly based on national wealth. Instead, they were split into two, strictly separating both the funding and the distribution of vaccines bought by rich and poor countries. HICs and Higher Middle Income Countries (HMICs), were grouped into the so-called “COVAX Facility” where they make down-payments to the Facility’s portfolio and receive doses in return. COVAX’s 92 poorest members (LICs and LMICs), as well as small island economies eligible for support from the World Bank’s International Development Association (IDA), were grouped into a separate buyers’ club, called the Gavi Advance Market Commitment for COVID-19 Vaccines, or “Gavi AMC”. They also need to contribute financially to their purchasing pool, but most of their funds stem from official development assistance (ODA), philanthropic and private sector donations as well as some private investments. Rich countries in the COVAX Facility were no longer obliged to provide direct financial assistance to the Gavi AMC. As Gavi’s CEO emphasized “the AMC is in no way cross-subsidised by the funds of self-financing participants” [1]. Instead HICs and HMICs were promised “a ring-fenced proportion [ … of vaccines, to be used] according to the guidance provided by their national bodies” [55]. The WHO’s carefully calibrated vaccine allocation framework may thus not have applied across the two buyers’ and distribution clubs (see Fig. 1).

### Supporting the pharmaceutical industry

COVAX’s second main goal was to support global vaccine production. Pharmaceutical companies have traditionally been held to underinvest in vaccines for developing countries, leading global vaccine development and manufacturing to be slower, less diverse and less widespread than they otherwise could be. Seen through the lens of risk, COVAX documents presented them as “too risk averse” to increase investments in essential pharmaceutical products, as they fear “development risk, demand risk and competition risk – all critical factors affecting the potential return on their investments in R&D and capacity establishment” [55]. COVAX focuses on these corporate risks that may arise in the pursuit of profit with a financial mechanism, by subsidizing pharmaceutical companies. This is done in two ways.

Firstly, COVAX provides “push incentives”, subsidizing company costs directly by paying with contributions of self-funding countries or with aid for pharmaceutical R&D and manufacturing capacity. For example, the Gates Foundation has provided $150 m to Gavi, which in turn passed those on to the Serum Institute of India, providing it with “upfront capital” to help it increase manufacturing capacity for AstraZeneca and Novavax vaccines, even before regulatory approval and WHO prequalification had been obtained [56]. Secondly, COVAX provides “pull incentives”, i.e. monetary support that is used to increase vaccine demand. For example, COVAX asks lower income countries to take regulatory steps that enable vaccination approval, to submit national deployment and vaccination plans, to hire and train vaccination staff, to prepare monitoring and data management tools as well as physical distribution capabilities (including transport and national cold chains), and to engage in social mobilisation to convince the public that future vaccination efforts will be in their interest. In doing so, COVAX turns developing countries from unlikely markets for vaccine sales into likely ones.

The most important tool for increasing vaccine demand, however, is known as Advanced Market Commitments (AMCs). COVAX’s AMCs are sets of contracts that go beyond standard advance purchase agreements in two main ways. Firstly, they provide individual vaccine developers and manufacturers with “volume guarantees” for vaccines before these are licensed. In this way, manufacturers know that they will not be outcompeted if and when their final product will be ready to be sold on the market. Secondly, they commit to market-wide demand guarantees available to any manufacturer, essentially committing to buying an overall quantity of vaccines if and when they are ready [56, 57].

AMCs were first developed by Gavi. Set up as a financing organisation with the goal of introducing and scaling up new and underused vaccines, Gavi soon endorsed a vaccine procurement system that was less focused on forcing down prices through single tender bulk purchasing, and favoured industry support [58]. As a document by Mercer Management Consulting, annexed to the minutes of Gavi’s first Board Meeting in 1999 argued, Gavi was seen to be operating in a market where a small number of pharmaceutical companies dominated vaccine production and would not change behaviour unless that promised increased profits. Mercer thus advised that Gavi strike a balance between the desire of country governments for low prices, and that of vaccine producers for high prices [58].

Gavi began to focus on corporate risk reduction soon thereafter as part of spending on rotavirus and pneumococcal vaccines in 2002. It first used “Accelerated Development and Introduction Plans (ADIPs)” to provide industry and developing country support, before working on AMCs. AMCs were developed by a working group that included members of the Gates Foundation, the World Bank and the Washington-based think tank Centre for Global Development (CGD) [59]. The group comprised economist Michael Kremer who is widely credited for coming up with the idea of AMCs, and who at the time held the title of “Gates Professor of Developing Societies” at Harvard University, funded by a Gates Foundation endowment [60]. The resulting report, “Making Markets for Vaccine: Ideas into action”, published in 2005 argued that public funds should be committed in advance to buying vaccines that were needed in developing countries, if and when they were developed. Governments and donors would pledge to pay a pre-set price and quantity for a fixed number of future vaccines. They would also pledge to top up low-income country vaccine payments. In return for such advanced commitments, pharmaceutical companies would produce the vaccines in question and agree to selling at a “low, fixed, sustainable” price to developing countries after the initial commitment was exhausted [59].

AMCs were explicitly promoted as a bulwark against changing intellectual property regimes. They address vaccine access issues “unlike many alternative proposals, [ …] without weakening incentives or dismantling the system of intellectual property rights” [59]. The CGD report cites private sector executives calling the potential discovery of a vaccine for AIDS their “worst nightmare” as they “would be forced to give it away” [59]. The report also emphasized that vaccines should not be too cheap as “the short-term need to get vaccines to many people competes with the long-term need to ensure that firms can meet the costs of R&D and also provide returns to shareholders” [59]. Lastly, the report mentions as a main AMC benefit to industry that it “significantly reduces the risk that, if a life-saving health product is invented, it will be subject to compulsory licensing, or that the firm will be forced to sell it at a loss, either because of the pressure of public opinion or because of the purchasing power of public procurement” [59].

AMCs were first put into practice in 2007 when the Gates Foundation and five countries pledged $1.5bn for an AMC funding pneumococcal conjugate vaccines (PCV). The AMC was officially launched by Gavi 2 years later, paying GlaxoSmithKline (GSK) and Pfizer $3.50 per dose for 30 million PCV doses each. Via subsequent tenders, prices sank to $2.90 and may soon reach $2 per dose [60, 61]. Defenders of the PCV’s subsidies claim that its $1.5bn have saved 700 k lives so far by increasing the available amount of PCVs. Whether this effort provided value for money is unclear, as the manufacturing costs of the PCV for GSK and Pfizer remain confidential. In spite of this uncertainty, AMC defenders argue that the corporate subsidies are worth it, as PCV doses bought via the AMC are far cheaper than those bought in high-income markets, and because AMC subsidies should not just aim to meet manufacturing costs, but pay companies a “reservation value”, i.e. the minimum price that pharmaceutical companies are willing to accept [61].

Whether such corporate subsidies can be justified depends to a large extent on their contractual details. AMCs are highly complicated contracts and can quickly turn from a reasonable subsidy of the private sector, to a perfect waste of aid [62]. A recent analysis of Gavi’s first AMC by Doctors without Borders for example, argues that it amounted to a mostly ineffective subsidy to Pfizer, GSK and the Indian Serum Institute, as it failed to speed up R&D, did not increase competition among manufacturers, may have fostered excess vaccine demand, did not lead to technology transfer to developing countries and lacked transparency [63]. What can be said with certainty is that after ten years of subsidies vaccination rates have risen significantly, prices have come down, and most of the $1.5bn went to two Western companies that have historically dominated vaccine production markets [64].

In the case of COVAX, contractual details that determine how big these subsidies have been mostly kept secret. While UNICEF and Gavi assure the public that the vaccines COVAX buys cost a mere $1.66 per dose [65], hardly any information about vaccine manufacturing costs, advance payment contracts or “push” and “pull” subsidies have been made public. COVAX’s vaccine delivery schedules and delivery enforcement mechanisms are also largely unknown. Leaked and haphazardly published information on vaccine prices suggests that vast differences are being paid globally for COVID-19 vaccines, with South Africa paying more than double per AstraZeneca dose ($5.25) than the European Union ($2.15) and Israel paying around double for each Pfizer-BioNTech dose compared to the EU [66]. Whether COVAX really receives not-for-profit prices for vaccines could only be assessed if manufacturing costs of COVID-19 vaccines were public knowledge. Legal liability in case vaccines have adverse effects due to corporate negligence or recklessness also lies with COVAX’ member countries [67].

## Discussion

### The buyers’ and distribution club

COVAX is part of long standing concerns over the unequal distribution of vaccines between rich and poor countries. These had already escalated during the spread of the avian influenza (H5N1). In 2006, 2 years after avian influenza had emerged, an Australian company had used virus samples from WHO’s publicly available Global Influenza Surveillance Network (GISN) to develop an avian influenza vaccine and was seeking patents for it. It was operating without the knowledge or consent of the developing countries that had supplied virus samples to GISN in the first place and who would not be able to afford the resulting products. Indonesia had been heavily affected by avian influenza and with the support of several developing countries, protested against vaccine inequity by refusing to share H5N1 virus samples via GISN. The country’s refusal, and its insistence on a connection between sharing influenza viruses and gaining access to resulting vaccines, ultimately forced the WHO to work towards greater vaccine equity [68].

As the interviewee quoted at the outset of the "Results" section of this paper mentions, similar concerns arose again in 2009 when the H1N1 strain of influenza emerged and rapidly spread. At the time, wealthy countries placed large advance orders for the corresponding vaccine, buying up most of its supply. While WHO and UN agencies collected monetary donations for developing countries to buy vaccines as well, these donations remained insufficient, leaving developing countries with limited supplies. Wealthy countries have since tended to block attempts to create legally binding arrangements that would ensure globally equitable vaccine access. This refusal has created a situation where only compromise solutions to work towards vaccine equity remained possible, shifting the focus of global health equity initiatives away from legally binding mechanisms towards non-binding ones, such as PPPs [69].

COVAX is one such PPP, a compromise solution between the world’s rich and poor countries, put together at a scale and scope never seen before [35]. Like other PPPs, it attempts to mediate power struggles between heterogeneous members within a single institutional framework, while trying not to lose its overarching goal of global vaccine equity out of sight. However, in order to accommodate for the divergent interests of its members, COVAX did not insist on the idea of globally shared health risk during this pandemic. Instead, it substantially weakened its governance structure, something that high-income countries took full advantage of. They struck a number of successful bilateral deals with vaccine companies, often for the same vaccines on which COVAX was relying, thereby outcompeting the PPP as part of buying up both future and actual vaccine doses. Deliveries on such bilateral deals outpaced COVAX procurement in scale and scope, as pharmaceutical companies prioritized countries that gave them the best deals. In early 2021 this included the UK, which sped up vaccine approval, Chile, which negotiated early and participated in clinical trials for several major vaccines [70], Israel, which paid higher prices per vaccine dose, bought large volumes, and allowed Pfizer to gather real-world data about the populations it vaccinated [71], and the USA, which worried less about vaccine cost or corporate liability for side effects [72].

The problem that COVAX would be undermined and that the world’s richest countries were buying up vaccines, often far beyond their needs, was already evident in late 2020. In December of that year Gavi published “Principles for sharing COVID-19 vaccine doses with COVAX”, effectively acknowledging that the buyers’ club had failed and creating a new role for COVAX by arguing that any country’s “excess doses” should be shared through the PPP [73]. COVAX’s target budget had been adjusted down by $4.3bn as money for self-financing countries was no longer required [49]. With the vaccination threshold for HICs and HMICs also having been adjusted from 20 to 50%, COVAX had changed from a buyers’ club based on global solidarity and sacrifice, to a charity-based aid project. While COVAX’s rhetoric continued to insist on a uniformly shared health risk between rich and poor countries, its structure suggests that said risk was deemed to be at least partially separable.

### Supporting the pharmaceutical industry

Regarding industry support, the creation of COVAX explicitly expanded Gavi’s corporate subsidy logic from developing countries with limited purchasing power to the world at large. This expansion came with an important shift in purpose, as the original AMC premise that global demand for vaccines may be insufficient or overly uncertain did not hold for COVID-19. Even in early 2020, when COVAX was set up, the possibility that pharmaceutical companies would *not* engage in research and development for COVID-19 vaccines was likely zero. BioNTech and Moderna had already begun SARS-CoV-2 vaccine research as early as January 2020 [74]. Thus the goal of subsidizing pharmaceutical companies shifted from a lack of demand, to decreasing the risk of ending up without vaccines by upping the speed, scale and scope of vaccine development. COVAX thereby reflected a global trend of moving biomedical research funding “downstream”, from backing basic research that is often conducted at universities, to supporting late stage research, clinical trials, development and manufacturing mostly carried out by corporations [75].

Expanding Gavi’s concern with corporate financial risks to the world at large does ignore its flipside, namely that most vaccine production had already been funded publicly for decades [76, 77] and that such financial risk is usually compensated internally by the pharmaceutical industry via substantial profits on successful products. Internal corporate risk mitigation enabled leading pharmaceutical companies before covid to earn net income margins (as a percentage of revenue) of 16.2%, far outperforming the profitability of any other subsection of companies listed on the S&P 500 [78]. Pfizer has earned $3.5bn in revenues from COVID-19 vaccines in just the first 3 months of 2021, with profit margins on the vaccine lying in the high 20% range [79]. In 2018, the 27 largest pharmaceutical companies held financial reserves worth $219bn, while the 10 largest of them held liquid assets worth $135bn. Most importantly, pay-outs to shareholders by major pharmaceutical companies (i.e. dividends and share buybacks) have grown from around 88% of total investments in 2000, to around 123% in 2018 [16]. COVAX’s premise that greater financial support for pharmaceutical companies is needed in the pursuit of vaccine equity downplays the existing public funding for vaccine development and ignores the ongoing decline in pharmaceutical corporate investment.

COVAX’s limited success over the first year and a half of its existence soon became obvious. By early August 2021, it had only delivered around 177 m vaccine doses [80], i.e. less than 10 % of the $2bn it had originally planned for the end of 2021. High and upper middle income countries had by then vaccinated around 60% of their populations with at least one dose, while only 1.8% of people in low income countries had received at least one COVID-19 vaccine dose [81]. Various factors have slowed the global vaccine rollout down. They include corporate supply shortages, a lack of regionally distributed production capacity, the unwillingness of pharmaceutical companies to voluntarily share vaccine IP through the WHO’s CTAP mechanism, and a refusal on the side of the EU Commission and Germany to support a temporary waiver of several sections of the World Trade Organization’s Agreement on Trade-Related Intellectual Property Rights (TRIPS). Yet, little evidence exists to suggest that corporate risk aversion, or a lack of available funds on the side of pharmaceutical companies have been major factors in these developments. Instead, past and present opposition between the use of AMCs and a softening of intellectual property regimes, as well as statements by members of COVAX’s governing institutions that challenging existing IP regimes is unnecessary or counterproductive [82] suggest that when in doubt, COVAX errs on the side of private sector finance. Its insistence to conflate corporate financial risk with global health risk may be partially to blame.

## Conclusion

This article has shown that COVAX is based on the recognition that a free market system is unlikely to ensure globally equitable vaccine access during a pandemic. Instead, COVAX uses buyers' and distribution club and various subsidies to the pharmaceutical industry to enhance the speed, scale and scope of COVID-19 vaccine production and distribution. To unite heterogenous parties into a single institutional effort, COVAX has relied on the notion of global public health risk. By systematically foregrounding financial risks of the pharmaceutical industry COVAX has shifted our understanding of public health risk from the people who may catch COVD-19, to the corporate intermediaries involved in producing vaccines. Its financialised approach to risk mitigation has not worked so far, as COVAX failed to avoid an international scramble for vaccines, has not brought about global vaccine equity, and cannot account for how it uses the vast amounts of aid money it has received. Its focus on corporate risk mitigation may be partially to blame for COVAX’s refusal to consider or support health equity policy measures that question or challenge corporate IP privileges. COVAX thus ends up constituting a risk itself. By perpetuating the downsides of the financialisation of global health, and by being used as a buffer against alternative global health measures it risks losing health equity out of sight in favour of equity markets.

## Data Availability

Not applicable.
